# Radiological Association Between Multiple Sclerosis Lesions and Serum Vitamin D Levels

**DOI:** 10.7759/cureus.31824

**Published:** 2022-11-23

**Authors:** Ali Akhtar, Rajiv Neupane, Amandeep Singh, Maham Khan

**Affiliations:** 1 Internal Medicine, National University of Medical Sciences, Rawalpindi, PAK; 2 Surgery, Nobel Medical College Teaching Hospital, Biratnagar, NPL; 3 Internal Medicine, University Hospital Coventry and Warwickshire, Coventry, GBR; 4 Radiology, Armed Forces Institute of Radiology and Imaging, Rawalpindi, PAK

**Keywords:** mri imaging, radiological evaluation, association, vitamin d, multiple sclerosis

## Abstract

Introduction: The primary aim of this study was to determine a plausible association between the radiological location of multiple sclerosis (MS) lesions and serum 25-hydroxyvitamin D (vitamin D) levels at the time of diagnosis. MS is a common immune-mediated neurological condition mainly affecting the central nervous system. Although the association of vitamin D levels is well established, there have not been many studies to propose a connection between the location of the lesions based on serum vitamin D levels. In this study, we determine the association between serum 25-hydroxyvitamin D and the radiological distribution of lesions in patients with MS.

Methods: Twenty patients with a confirmed diagnosis of MS involving new T2-weighted and gadolinium-enhancing T1-weighted lesions in the entire central nervous system (brain and spinal cord) with serum 25-hydroxyvitamin D levels at the time of diagnosis were included in a case group. As a reference, 20 patients with a confirmed diagnosis of MS with isolated new T2-weighted and gadolinium-enhancing T1-weighted lesions (either supratentorial, infratentorial, or spinal cord) with serum 25-hydroxyvitamin D levels at the time of diagnosis were included in the control group.

Results: The mean serum 25-hydroxyvitamin D level was significantly low in the case group compared to the control group (36.2 ± 17.2 vs 62.6 ± 21.0; p-value <0.0001).

Conclusion: There is a plausible inverse relationship between serum vitamin D and the MS lesions involving the entire central nervous system (brain and spinal cord). This evidence may enable clinicians to forecast disease load based on serum vitamin D levels.

## Introduction

Multiple sclerosis (MS) is a disorder of the central nervous system which targets the myelin sheath causing inflammation and leading to demyelination and degenerative changes. In the United Kingdom, a little more than 100,000 people are living with MS which is considered the most common non-traumatic cause of long-term neurological disability in patients aged less than 40 years [1,2]. The age of onset is usually between 20 and 50 years, affecting females in two-thirds of the cases [1]. Magnetic resonance imaging (MRI) is often the chosen modality to radiologically support the diagnosis of MS. However, there are certain conditions (e.g. neuromyelitis optica spectrum disorders and Susac syndrome) that can also satisfy the MRI criteria for MS, thus making it challenging to diagnose MS [1,2]. To differentiate between these conditions, we often seek biochemical tests to confirm the diagnosis (e.g. oligoclonal bands in serum and cerebrospinal fluid (CSF) paired to evaluate MS). Several studies have implicated lower levels of serum 25-hydroxyvitamin D (serum vitamin D) contributing to increasing relapses in MS patients [3,4]. However, there is only trace evidence of actual radiological mapping of lesions based on the location in patients with hypovitaminosis D. 

## Materials and methods

This retrospective case-control study was conducted in a neurosciences department in a tertiary care hospital (University Hospital Coventry and Warwickshire NHS Trust, United Kingdom) from 2021 to 2022. Fifty-four participants were included at the beginning of the study. Fourteen participants were excluded from the study due to the unavailability of MRI images or the uncertain history of vitamin D supplements. Twenty patients with a confirmed diagnosis of MS (based on the Mcdonald’s criteria, clinical symptoms, and the presence of oligoclonal bands in the CSF) involving new T2-weighted and gadolinium-enhancing T1-weighted lesions in the entire central nervous system (brain and spinal cord) with serum 25-hydroxyvitamin D levels at the time of diagnosis were included in a case group. As a reference, 20 patients with a confirmed diagnosis of MS (based on the Mcdonald’s criteria, clinical symptoms, and the presence of oligoclonal bands in the CSF) with isolated new T2-weighted and gadolinium-enhancing T1-weighted lesions (either supra tentorial, infratentorial, or spinal cord) with serum 25-hydroxyvitamin D levels at the time of diagnosis were included in the control group. The MS-specific lesion(s) was mapped based on their location as the supratentorial, infratentorial, spinal cord, or the entire central nervous system (brain and spinal cord). The supratentorial lesion(s) was defined as one or more lesions involving the parts of the brain above the tentorium cerebelli (e.g. cerebrum, periventricular areas, pineal gland, hypothalamus, pituitary gland, and optic nerve). Infratentorial lesion(s) was defined as one or more lesions involving the parts of the brain below the tentorium cerebelli (e.g. cerebellum, tectum, fourth ventricle, midbrain, pons, and medulla). The spinal cord lesion(s) was defined as one or more lesions located at any level of the spinal cord (e.g. cervical, thoracic, lumbar, and sacral). The central nervous system lesion(s) was defined as one or more lesions involving the entire brain (e.g. supratentorial and infratentorial) and spinal cord (e.g. cervical, thoracic, lumbar, and sacral). Serum 25-hydroxyvitamin D levels of less than 50 nmol/l were considered hypovitaminosis D. 

Brain and spinal cord MRI images were taken using a 3T Philips MR 7700 scanner (Philips, Amsterdam, Netherlands). Radiologist reports were reviewed alongside images (using picture archiving and communication system) by an expert panel (blinded from serum vitamin D levels) at baseline. T1, T2, and FLAIR/STIR sequences were obtained. Gadolinium-enhanced T1-weighted images were also acquired five minutes after administering a single dose (0.1mmol/kg) of contrast agent.

Statistical analyses were performed using IBM SPSS Statistics for Windows, Version 25 (Released 2017; IBM Corp., Armonk, New York, United States). Age, duration of disease, and mean serum 25-hydroxyvitamin D levels were continuous variables and, hence, were presented as mean and standard deviation. The categorical variables including gender (female), MS subtype, and hypovitaminosis D were presented as percentages and frequencies. For numerical values, an independent T-test was applied to compare the two groups. For categorical data, a Chi-square test was applied. A p-value of less than 0.05 denoted a significant difference between the case and control, and the null hypothesis was void.

## Results

A total of 54 participants with a confirmed diagnosis of MS were studied. Out of these, 14 were excluded due to either unavailability of MRI image sequences or uncertain vitamin D history. The mean serum 25-hydroxyvitamin D levels were significantly low in MS patients with new lesions involving the entire central nervous system (brain and spinal cord) as compared to the control group where only isolated (either supratentorial, infratentorial, or spinal cord) lesions were present (36.2 ± 17.2 nmol/l vs 62.6 ± 21.0 nmol/l; P-value <0.0001). This signifies an inverse relationship between serum vitamin D levels and the heavy distribution of MS lesions involving the entire central nervous system. Similarly, the number of participants with hypovitaminosis D having lesion(s) involving the central nervous system (brain and spinal cord) was significantly more in the case group as compared to the control group (n=14 vs n=6; P-value <0.05). 

The mean age of participants in the case group was 33.9 ± 12.4 years, while that in the control group was 35.0 ± 10.7 years. The majority of cases were of relapsing-remitting MS in both groups, 95% (n=19) in the case group and 90% (n=18) in the control group. Primary progressive MS was 5% (n=1) and 10%(n=2) in the case and control groups, respectively. The differences between gender, time since the duration, age, and disease subtype (e.g. relapsing-remitting and primary progressive) were statistically non-significant (Table 1).

**Table 1:** Baseline characteristics SD, standard deviation; MS, multiple sclerosis; RRMS, relapsing-remitting multiple sclerosis; PP, primary progressive

Characteristics	Case group (n=20)	Control group (n=20)	P-value
Age (mean ± SD)	33.9 ± 12.4 (years)	35.0 ± 10.7 (years)	0.77
Time since duration (mean ± SD)	3.43 ± 2.93 (years)	4.11 ± 4.9 (years)	0.59
Female (%)	14 (70)	14 (70)	1.00
Mean serum vitamin D levels (mean ± SD)	36.2 ± 17.2 (nmol/l)	62.6 ± 21.0 (nmol/l)	<0.0001
Hypovitaminosis D (%)	14 (70)	6 (30)	<0.05
MS subtype - RRMS (%)	19 (95)	18 (90)	0.55
MS subtype - PP (%)	1 (5)	2 (10)	0.55

## Discussion

Our study highlights the correlation between serum 25-hydroxyvitamin D and the radiological distributions of MS lesions. From the data, we can deduce that hypovitaminosis D is associated with disease load spanning across the entire central nervous system (brain and spinal cord) as compared to the isolated distribution of lesions (e.g supratentorial, infratentorial, or spinal cord). This association was robust in our study. It is worth noting that new T2 lesions involving individual segments (e.g. either supratentorial, infratentorial, or spinal cord) have had normal mean serum 25-hydroxyvitamin D levels. Most of the lesions in these patients were located in either supratentorial or at the spinal cord level.

A cohort almost a decade ago showed an inverse relation of serum vitamin D levels to MS activity on MRI brain [5]. The study also claimed the association between new lesions of MS and a later disability as assessed by the expanded disability status scale (EDSS). The association of serum vitamin D with clinical relapses was less significant, owing to some participants being closely followed up by neurologists [5]. Another study by Rito et al. did not show any significant link between the EDSS scale and serum vitamin D [6].

A previous study has shown a connection between the major histocompatibility complex (MHC) class II allele HLA-DRB1*1501 (dominant haplotype of northern Europe) and the risk of MS by threefold. This MHC on chromosome 6 has a strong genetic linkage as well [6]. The environment also plays a crucial role in the development of MS, and there are a number of studies which now support the geographical distribution and involvement of vitamin D influencing the risk of MS [5,7]. Vitamin D plays a significant role in the immune and nervous systems. In this study, it was found that vitamin D interacts with HLA-DRB1*1501 and influences its expression [7].

The role of smoking has also been explored in previous studies. Smoking has been associated with the development of new T2 lesions and clinical relapses. This could be another potential confounding factor in our study. However, the association of smoking with disease progression has not been well established [5].

Our study has a few limitations. First, our study did exclude patients taking oral vitamin D supplements but despite this, we were unable to exclude patients who were already taking food supplemented with vitamin D (e.g. fortified food). Second, we had a small sample size as not all participants underwent complete sequences of the brain and spinal cord images together at the time of diagnosis. Therefore, we propose that the study should be replicated on a wider scale to further determine the association between MS lesions and vitamin D levels.

## Conclusions

In this study, we found that low levels of serum vitamin D were associated with MS lesions involving the entire central nervous system (brain and spinal cord). This inverse relationship based on radiological evidence can reinforce the importance of vitamin D in the management of MS. We suggest that further studies should be performed on a wider scale to enable clinicians to forecast MS lesions based on serum vitamin D levels.
